# Disparities in treatment patterns and mortality in prostate cancer: Interaction between Black race and end‐stage kidney disease

**DOI:** 10.1002/cam4.7027

**Published:** 2024-05-21

**Authors:** Nagaraju Sarabu, Weichuan Dong, Siran M. Koroukian

**Affiliations:** ^1^ Division of Nephrology Department of Medicine, The Metrohealth System Cleveland Ohio USA; ^2^ Department of Medicine Case Western Reserve University School of Medicine Cleveland Ohio USA; ^3^ Population and Quantitative Health Sciences, Population Cancer Analytics Shared Resource, and the Case Comprehensive Cancer Center Case Western Reserve University Cleveland Ohio USA

**Keywords:** Black race, end‐stage kidney disease, mortality, prostate cancer, racial disparity, treatment

## Abstract

**Background:**

Black men and men with end‐stage kidney disease have lower rates of treatment and higher mortality for prostate cancer. We studied the interaction of end‐stage kidney disease (ESKD) with Black race for treatment rates and mortality for men with prostate cancer.

**Methods and Results:**

We included 516 Black and 551 White men with ESKD before prostate cancer 22,299 Black men, and 141,821 White men without ESKD who were 40 years or older from the Surveillance, Epidemiology, and End‐Results‐Medicare data (2004–2016). All Black men with or without ESKD and White men with ESKD had higher prostate‐specific antigen levels at diagnosis than White men without ESKD. Black men with ESKD had the lowest rates for treatment in both local and advanced stages of prostate cancer (age‐adjusted risk ratio: 0.76, 95% Confidence Interval (CI): 0.71–0.82 for local stage and age‐adjusted risk ratio: 0.82, 95% CI: 0.76–0.9 for advanced stages) compared to White men without ESKD. Compared to White men without ESKD, prostate cancer‐specific mortality was higher in White men with ESKD for both local and advanced stages (age‐adjusted hazard ratio: 1.8, 95% CI: 1.2–2.8 and HR: 1.6, 95% CI: 1.2–2.2) and it was higher for ESKD Black men only in advanced stage prostate cancer (age‐adjusted hazard ratio: 2.4, 95% CI: 1.5–3.6).

**Conclusion:**

Our findings suggest that having a comorbidity such as ESKD makes Black men more vulnerable to racial disparities in prostate cancer treatment and mortality.

## Graphical abstract

Men with end‐stage kidney disease (ESKD) present with higher prostate specific antigen at the time of prostate cancer. An interaction between Black race and ESKD was noted for receipt of treatment and prostate cancer‐specific mortality as Black men with ESKD before cancer diagnosis had the lowest probability of receiving treatment and highest prostate cancer‐specific mortality, compared to White men without ESKD. If men with prostate cancer have a severe comorbidity such as ESKD, more intense efforts are needed to reduce racial disparities.

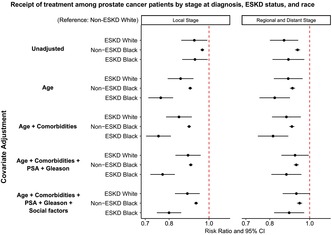

## 1 INTRODUCTION

Racial and ethnic healthcare disparities are defined as racial or ethnic differences in the quality of healthcare that are not due to access‐related factors or clinical needs, preferences, and appropriateness of intervention.^1^ The prevalence of prostate cancer and end‐stage kidney disease (ESKD), which is defined as being maintained on dialysis or having a kidney transplant, in men in the United States is as follows: over 3 million men with prostate cancer and 2769 men per million with ESKD lived in 2020.^2^, ^3^ In a recent report of the United States national ESKD registry, it was shown that ESKD men have a prevalence of 3.1% for prostate cancer based on Medicare claims and the percentage of men with ESKD who were Black was higher in those who had prostate cancer than those without (39% vs. 27%).^4^ Racial healthcare disparities for Black men exist for both diseases. Black men with prostate cancer experience 1.78‐fold higher incidence and 2.2‐fold higher mortality compared to White men.^5^ Many mechanisms, including inherited susceptibility of genes involved in prostate cell and tissue repair (BRCA1/2, ATM), regeneration (HOXB13 and MYC), diet and lifestyle, environmental factors that promote prostate inflammation, lower quality and accessibility of health care, and structural and institutional racism have been proposed to be responsible for this disparity in prostate cancer outcomes for Black men.^6^, ^7^ Health disparities are also evident in Black people with ESKD. In the United States, Black people account for 13.6% of the population.^8^ However, more than 30% of the ESKD patients are Black with ESKD prevalence being four times higher among Black people than White people.^9^ Interestingly, Black people with ESKD were shown to have lower mortality compared to White people, despite healthcare disparities in dialysis delivery, arteriovenous fistula rates, and home dialysis rates among.^10^, ^11^, ^12^

In low‐ and intermediate‐risk local prostate cancer, it was recently shown that older (>66 years) patients with ESKD, had 5‐fold higher mortality than those without ESKD, and received nearly similar rates of definitive treatment (prostatectomy or radiation).^13^ How racial disparities behave when prostate cancer and ESKD co‐occur is unclear. We hypothesized that the interaction of Black race and ESKD would exaggerate the healthcare disparities for treatment and prostate cancer‐specific mortality.

## 2 METHODS AND RESULTS

We identified men who were 40 years of age or older and diagnosed with invasive prostate cancer during 2004–2016 from Surveillance, Epidemiology, and End‐Results (SEER)‐Medicare data which included patients from the states of California, Connecticut, Georgia, Hawaii, Iowa, Kentucky, Louisiana, New Jersey, New Mexico, and Utah, and the metropolitan counties of Detroit and Seattle Puget Sound. The Case Western Reserve University Institute Review Board approved the study with a waiver of informed consent (Protocol #2019‐0264).

Our independent variables were ESKD status before prostate cancer diagnosis and race (Black vs. White). ESKD status was determined using billing codes (see supplement) as described previously,^13^ and race was collected by the SEER from various sources, including patient reports and medical records. We identified comorbidities from the Medicare beneficiary summary file from the middle of the year of diagnosis of prostate cancer. We used non‐ESKD White men as the reference group in all regression models. Receipt of treatment was defined as surgery or radiation for the local stage and surgery or radiation or chemotherapy or androgen deprivation therapy for regional and distant stages. We used the “Summary‐Stage‐2000” variable for ascertaining the cancer stage. Stage was defined as local if the cancer is limited to the prostate gland, regional if cancer has spread to nearby lymph nodes, tissues, or organs, and distant if cancer has spread to distant parts of the body. Surgery or radiation was obtained from SEER directly and the rest of the treatments were ascertained using billing codes (Supplement).

We conducted multivariable robust Poisson regression analysis for receipt of treatment and Fine and Gray competing risk analysis for overall and prostate cancer‐specific mortality.^14^ Robust Poisson regression model, which provides a robust standard error estimate, and estimated covariate effects interpretable as risk ratios, is a valid approach for a binary outcome as noted in the literature.^15^ All analyses were conducted separately for the local stage and for the regional and distant stages combined.

Selection of the study participants based on inclusion and exclusion criteria is illustrated in Figure S1. Among men who were diagnosed with local prostate cancer, 413 were Black, 402 were White who had ESKD before prostate cancer, 19,183 were Black, and 120,008 White who did not have ESKD. Among men who were diagnosed with regional or distant stage prostate cancer, 103 were Black, 149 were White who had ESKD before prostate cancer, 3116 were Black, and 21,813 White who did not have ESKD. Among those with local stage prostate cancer, Black men with ESKD were the youngest and White men without ESKD were the oldest. In the ESKD group, compared to White men, Black men were less likely to be married, to live in a census tract with ≤20% of the population below the federal poverty level, and more likely to live in a metropolitan county, and had similar comorbidities except for higher hypertension rate. ESKD Black men, ESKD white men, and non‐ESKD Black men had higher rates of PSA >20 than non‐ESKD White men. Similar patterns of baseline characteristics were observed in the regional and distant‐stage prostate cancer subjects (Table 1).

**TABLE 1:** Baseline characteristics of patients diagnosed with invasive prostate cancer from the Surveillance, Epidemiology, and End‐Result‐Medicare data, 2004–2016, stratified by cancer stage, end‐stage kidney disease status and race (Black/White).

Patients diagnosed with local stage prostate cancer						
	ESKD Black (*N* = 413)	ESKD White (*N* = 402)	*p*‐value	Non‐ESKD Black (*N* = 19,183)	Non‐ESKD White (*N* = 120,008)	*p*‐value
Age at cancer diagnosis (mean (SD))	65.1 (8.7)	70.2 (8.7)	<0.001	70.4 (7.4)	73.2 (6.4)	<0.001
Marital status						
Married/Partnered	192 (46.5)	230 (57.2)	0.009	9539 (49.7)	80,110 (66.8)	<0.001
Single	162 (39.2)	125 (31.1)		6840 (35.7)	22,635 (18.9)	
Unknown	59 (14.3)	47 (11.7)		2804 (14.6)	17,263 (14.4)	
Living in a census tract with ≤20% population under the federal poverty	208 (50.4)	314 (78.1)	<0.001	9822 (51.2)	102,496 (85.4)	<0.001
Living in Metropolitan county	379 (91.8)	336 (83.6)	<0.001	17,000 (88.6)	98,780 (82.3)	<0.001
Cardiovascular disease	297 (71.9)	295 (73.4)	0.638	7354 (38.3)	51,538 (43)	<0.001
Hypertension	367 (88.9)	334 (83.1)	0.017	13,005 (67.8)	68,201 (56.8)	<0.001
Diabetes	256 (62.0)	236 (58.7)	0.339	6631 (34.6)	28,876 (24.1)	<0.001
Prostate specific antigen						
<10	227 (55.0)	177 (44.0)	0.015	10,847 (56.5)	74,797 (62.3)	<0.001
10–20	68 (16.5)	75 (18.7)		3225 (16.8)	17,209 (14.3)	
>20	53 (12.8)	63 (15.7)		2310 (12.0)	8517 (7.1)	
Unknown	65 (15.7)	87 (21.6)		2801 (14.6)	19,485 (16.2)	
Gleason score						
≤6	163 (39.5)	165 (41.0)	0.114	7430 (38.7)	50,088 (41.7)	<0.001
7	164 (39.7)	131 (32.6)		7619 (39.7)	44,711 (37.3)	
≥8	64 (15.5)	75 (18.7)		3121 (16.3)	18,709 (15.6)	
Unknown	22 (5.3)	31 (7.7)		1013 (5.3)	6500 (5.4)	
Received definitive treatment^a^	267 (64.7)	259 (64.4)	0.947	12,926 (67.4)	83,926 (69.9)	<0.001
Median follow‐up time^b^	3.7 years	3.3 years		6 years	6.4 years	
Total deaths during study period	236 (57.1)	261 (64.9)	0.023	6046 (31.5)	35,933 (29.9)	<0.001
Deaths due to prostate cancer	<11 (<2.7%)	24 (6.0)	0.007	880 (4.6)	4908 (4.1)	<0.001

Patients diagnosed with regional or distant stage prostate cancer						
	ESKD Black (*N* = 103)	ESKD White (*N* = 149)	*p*‐value	Non‐ESKD Black (*N* = 3116)	Non‐ESKD White (*N* = 21,813)	*p*‐value
Age at cancer diagnosis (mean (SD))	65.9 (11.2)	76.5 (9.5)	<0.001	70.9 (8.9)	73.9 (7.8)	<0.001
Marital status^c^						
Married/Partnered	56 (54.4)	81 (54.4)	0.792	1430 (45.9)	15,190 (69.6)	<0.001
Single	47 (45.6)	68 (45.6)		1469 (47.1)	5414 (24.8)	
Unknown				217 (7.0)	1209 (5.5)	
Living in a census tract with ≤20% population under the federal poverty	46 (44.7)	120 (80.5)	<0.001	1428 (45.8)	18,495 (84.8)	<0.001
Living in Metropolitan county	90 (87.4)	123 (82.6)	0.298	2803 (90.0)	17,972 (82.4)	<0.001
Cardiovascular disease	61 (59.2)	112 (75.2)	0.007	1106 (35.5)	8969 (41.1)	<0.001
Hypertension	79 (76.7)	127 (85.2)	0.085	1878 (60.3)	11,669 (53.5)	<0.001
Diabetes	44 (42.7)	64 (43.0)	0.971	860 (27.6)	4786 (21.9)	<0.001
Prostate specific antigen						
<10	18 (17.5)	17 (11.4)	0.248	856 (27.5)	8723 (40.0)	<0.001
10–20	14 (13.6)	18 (12.1)		398 (12.8)	3178 (14.6)	
>20	57 (55.3)	81 (54.4)		1505 (48.3)	7335 (33.6)	
Unknown	14 (13.6)	33 (22.2)		357 (11.5)	2577 (11.8)	
Gleason Score^c^						
≤6	31 (30.0)	24 (16.1)	0.050	213 (6.8)	1271 (5.8)	0.004
7				991 (31.8)	7294 (33.4)	
≥8	37 (35.9)	60 (40.3)		1244 (39.9)	8997 (41.3)	
Unknown	35 (34.0)	65 (43.6)		668 (21.4)	4251 (19.5)	
Received definitive treatment^a^	85 (82.5)	120 (80.5)	0.691	2706 (86.8)	20,267 (92.9)	<0.001
Median follow‐up time^b^	1.6 years	1.2 years		3.4 years	3.8 years	
Total deaths during study period	76 (73.8)	129 (86.6)	0.010	1649 (52.9)	9834 (45.1)	<0.001
Deaths due to prostate cancer	36 (35.0)	67 (45.0)	0.031	1009 (32.4)	5867 (26.9)	<0.001

^a^ For local stage disease, definitive treatment is defined as prostatectomy or radiation, or both; for regional and distant stage diseases, definitive treatment is defined as prostatectomy, radiation, androgen deprivation therapy, chemotherapy, or any combination of them.

^b^ Median follow‐up time (years) is calculated from the date of diagnosis to the date of death or end of study, whichever comes first.

^c^ Some rows collapsed due to low numbers in cells to maintain anonymity per SEER reporting guidelines.

**FIGURE 1:**
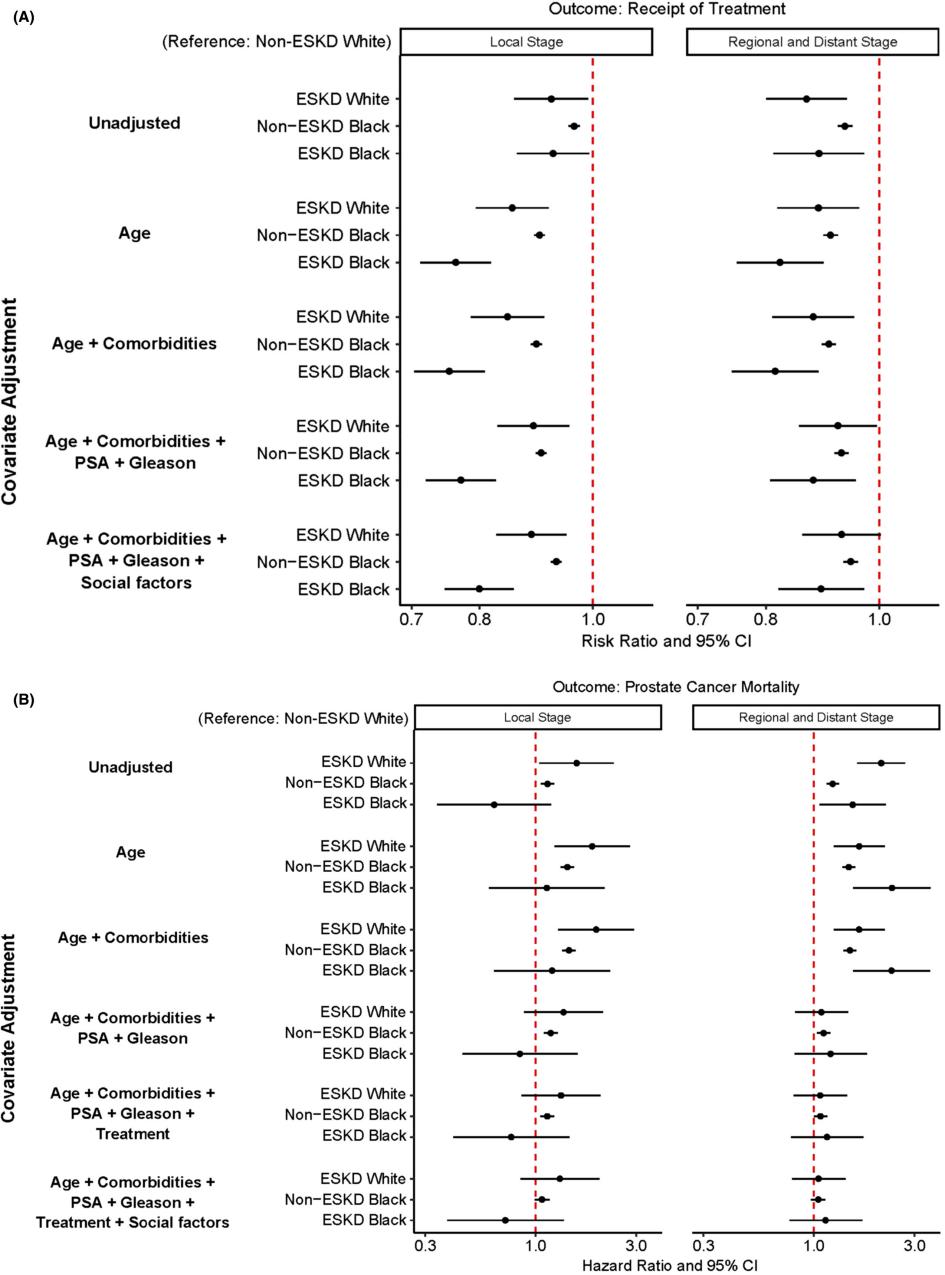
Outcomes of patients with invasive prostate cancer from the Surveillance, Epidemiology, and End‐Result‐Medicare data, 2004–2016, stratified by end‐stage kidney disease (ESKD) before prostate cancer diagnosis and Black or White race. (A) Receipt of definitive treatment by stage at diagnosis. (B) Prostate cancer‐specific mortality by stage at diagnosis. (1) For local stage prostate cancer, definitive treatment is defined as prostatectomy or radiation, or both; for regional and distant stage prostate cancer, definitive treatment is defined as prostatectomy, radiation, androgen deprivation therapy, chemotherapy, or any combination of them. (2) The x‐axis is on a logarithmic scale. (3) Comorbidities include cardiovascular disease, diabetes, and hypertension. (4) Social factors include individual‐level marital status, whether in a census tract with ≤20% of the population below the federal poverty level, and whether in a metropolitan county. (5) The confidence intervals for certain groups might be too narrow to visualize clearly. PSA, prostate‐specific antigen.

For local prostate cancer, after adjustment for age, there was a stepwise decrease in the receipt of treatment in non‐ESKD Black men (adjusted risk ratio (aRR): 0.90, 95% Confidence Interval (CI): 0.89–0.91), ESKD White men (aRR: 0.85, 95% CI: 0.79–0.92), and ESKD Black men (aRR: 0.76, 95% CI: 0.71–0.82), compared to non‐ESKD White men. This pattern was the same after further stepwise adjustments for comorbidity, PSA, Gleason score, and social factors (Figure 1A). Similarly, for regional and distant prostate cancer, non‐ESKD White men were more likely to receive treatment compared to all other groups. After adjustment for age, Black race and ESKD status were both associated with lower receipt of treatment. ESKD Black men were 18% less likely to receive treatment for advanced stages ESKD Black men (aRR: 0.82, 95% CI: 0.76–0.9), compared to non‐ESKD White men (Figure 1A).

At median follow‐up times of 3.7, 3.3, 6, and 6.4 years for local stage prostate cancer for ESKD Black men, ESKD White men, non‐ESKD Black men, and non‐ESKD White men, 57%, 65%, 32%, and 30% died respectively. Black men who had ESKD before prostate cancer had the highest hazards of death for both local stage (adjusted hazard ratio (aHR): 6.5, 95% CI: 5.7–7.4) and advanced stage (HR: 6.1, 95% CI: 4.9–7.7) compared to non‐ESKD White men (Figures S2 and S3). For prostate cancer‐specific mortality, in the local stage, ESKD White men had higher hazards (HR: 1.8, 95% CI: 1.2–2.8), non‐ESKD Black men also had higher hazards (aHR: 1.4, 95% CI: 1.3–1.5), but ESKD Black men had similar hazards (aHR: 1.1, 95% CI: 0.6–2.1), compared to non‐ESKD White men in age‐adjusted models. The higher hazards for ESKD White men dissipated after adjustment for PSA and Gleason score (Figure 1B). In the regional and distant stages, prostate cancer‐specific mortality was again highest for ESKD Black men (aHR: 2.4, 95% CI: 1.5–3.6), compared to non‐ESKD White men in the age‐adjusted model. This relative risk dissipated after adjustment for PSA and Gleason score (Figure 1B).

## 3 DISCUSSION

In this retrospective cohort study of Medicare beneficiaries with prostate cancer, we observed the following interesting findings: (1) Black men with or without ESKD before prostate cancer diagnosis and White men with ESKD before prostate cancer diagnosis had higher PSA at the time of diagnosis, compared to non‐ESKD White men. (2) Being diagnosed with ESKD before prostate cancer accentuated the racial healthcare disparity in receiving treatment for Black men. (3) Being diagnosed with ESKD before prostate cancer also accentuated the racial disparity in prostate cancer‐specific mortality for Black men. (4) These disparities for Black men with ESKD before prostate cancer were blunted after adjustment for PSA and Gleason score.

The fact that our study participants were all Medicare beneficiaries had two implications for our results. One, it provided equal healthcare coverage for all study subjects. Two, ESKD patients were younger due to their eligibility for Medicare even if they were younger than 65 years of age. Black men with ESKD were younger than White men with ESKD, which drove additional confounding of results due to age. In age‐adjusted models, Black men with ESKD before prostate cancer were 24% less likely to receive treatment for local prostate cancer and 18% less likely to receive treatment for regional and distant prostate cancer than White men without ESKD. A previous study comparing treatment patterns of local prostate cancer (categorized based on the National Comprehensive Cancer Network framework) found that ESKD patients with low‐risk prostate cancer have lower but those with intermediate‐risk prostate cancer have similar rates of prostatectomy or radiation therapy compared to those without ESKD.^13^ We expand on those findings by studying race disparities and including any stage of prostate cancer. Black men have consistently been shown to be less likely to receive treatment for prostate cancer than White men in the general population, which has been attributed to lack of insurance, poor access to healthcare, and low socioeconomic status but the racial disparity in treatment was not seen in equal access to health insurance such as the Veteran Affairs system or in clinical trials.^16^, ^17^, ^18^ The fact that Black men, especially those with ESKD before prostate cancer diagnosis, in our study had equal healthcare coverage and still were less likely to receive treatment, which persisted after adjustment for prostate cancer characteristics and social factors is intriguingly concerning.

It is well known that Black men have higher prostate cancer‐specific mortality than White men in the general population.^19^ It is again shown in our study that Black men without ESKD before prostate cancer had 42% and 47% higher prostate cancer‐specific mortality than White men without ESKD for local and advanced stages of prostate cancer respectively. Interestingly, while ESKD White men had higher prostate cancer‐specific mortality for any stage prostate cancer than non‐ESKD White men, Black men with ESKD had higher prostate cancer‐specific mortality than non‐ESKD or ESKD White men only for advanced stages. Black men with ESKD before prostate cancer having similar prostate cancer mortality in the local stage may be due to confounding by age or the inability of the study to detect differences due to a low number of prostate cancer‐specific deaths. The disparity in prostate cancer‐specific mortality for Black men with ESKD for advanced stages was eliminated after adjustment for PSA and Gleason score in the advanced prostate cancer stages, which may indicate that the aggressive nature of prostate cancer at presentation, which could be due to delayed diagnosis, was driving it. High‐risk disease at presentation and lower rates of treatment for Black men are well described in the general population.^20^, ^21^, ^22^

Our study has limitations. Since it is limited to Medicare fee‐for‐service beneficiaries and from states that participate in SEER, the findings may not apply to patients in managed care or other states or countries. Because men who were less than 65 years of age in our study would have been included only if they had ESKD or Medicare‐eligible disabilities, the non‐ESKD men in this age group were very different from the general population and the results need to be interpreted with caution. The social factors we assessed were narrow in scope to effectively assess social determinants of health as mediators of racial disparity in Black men with ESKD. SEER contains treatment for only the first round of treatment for radiation and it is known that sensitivity in detecting radiation is limited. However, we expect that the sensitivity for capturing radiation treatments would be similar across all the groups. The number of men with ESKD was low to detect small differences but using a national registry made it possible to achieve more numbers than single or multicenter studies.

In conclusion, under uniform fee‐for‐service Medicare coverage, having an additional major comorbidity such as an organ failure (ESKD) accentuates the racial disparity in access to treatment and prostate cancer‐related mortality for Black men. Healthcare facilities must intensify their efforts to achieve health equity for Black men with prostate cancer by understanding treatment decision factors if they also have additional major comorbidities such as organ failure. Further mixed methods research must confirm these findings and explore interventions to reduce the observed disparities.

## AUTHOR CONTRIBUTIONS

**Nagaraju Sarabu:** Conceptualization (equal); funding acquisition (equal); investigation (equal); methodology (equal); writing – original draft (lead). **Weichuan Dong:** Data curation (equal); formal analysis (equal); methodology (equal); writing – review and editing (supporting). **Siran M. Koroukian:** Project administration (equal); supervision (lead); writing – review and editing (supporting).

## FUNDING INFORMATION

Supported by intramural internal funding from the University Hospital of Cleveland Research Department. The University Hospital of Cleveland Research Department had no role in the design and conduct of the study; collection, management, analysis, and interpretation of the data; preparation, review, or approval of the manuscript; and decision to submit the manuscript for publication.

## CONFLICT OF INTEREST STATEMENT

Dr. Koroukian and Dr. Dong are supported by the National Cancer Institute, Case Comprehensive Cancer Center (P30 CA043703), and the American Cancer Society (132678‐RSGI‐19‐213‐01‐CPHPS), and by contracts from Cleveland Clinic Foundation, including a subcontract from Celgene Corporation. In the past 36 months, Dr. Koroukian was also supported by grants from the Centers for Disease Control and Prevention (U48 DP005030‐05S1 and U48 DP006404‐03S7); the National Institutes of Health (R15 NR017792, and UH3‐DE025487); the American Cancer Society (RWIA‐20‐111‐02 RWIA).

## ETHICS STATEMENT

The Case Western Reserve University Institute Review Board approved the study with a waiver of informed consent (Protocol #2019‐0264).

## Supporting information


Data S1.

## Data Availability

The data underlying this article were provided by Surveillance, Epidemiology, and End Results by permission. Data will be shared on request to the corresponding author with the permission of Surveillance, Epidemiology, and End Results.
