# Supplementary material for: Disparities in treatment patterns and mortality in prostate cancer: Interaction between Black race and end‐stage kidney disease
Source: Cancer Med. 2024 May 21;13(10):e7027. doi: 10.1002/cam4.7027 (PMC11106677; doi:10.1002/cam4.7027)

**Supplement Material:**

**ESKD patients include dialysis and kidney transplant patients:**

**Transplant patients:** Those who had a billing claim with kidney transplant-related codes (see below) in the 365 days window, before prostate cancer diagnosis, and did not have any dialysis claim after the kidney transplant.

**Dialysis patient:** Those who had a billing claim with chronic renal failure (CRF)-related codes (see below) and a billing claim with dialysis-related codes (see below) in the 365 days window, before prostate cancer diagnosis.

**ICD diagnosis codes**

CRF: any subcategories starting with ICD 9 codes: 582, 585, 586, 587, 403, 404, and 250.4 or ICD-10 codes N18.3, N 18.4, N18.5 and N18.6

Kidney Transplant: ICD 9: V420; ICD 10: Z940

**CPT/HCPCS codes**

**Dialysis:** 90918, 90919, 90920, 90921, 90922, 90923, 90924, 90925, 90935, 90936, 90937, 90945, 90946, 90947, 90951, 90952, 90953, 90954, 90955, 90956, 90957, 90958, 90966, 90967, 90968, 90969, 90970, 90971, 90972, 90973, 90974, 90975, 90976, 90977, 90978, 90979, 90980, 90981, 90982, 90983, 90984, 90985, 90988, 90989, 90990, 90991, 90994, 99559

**Kidney Transplant:** 50360, 50365

**ICD procedure codes**

**Dialysis:** 3995, 5498
**Kidney Transplant:**5569

**Revenue center codes**

**Dialysis:** any codes starting with 082, 083, 084, 085, 086, 087

**CPT/HCPCS codes for Androgen deprivation therapy or chemotherapy**

A9606, C9216, C9399, C9399, C9430, G0356, G9132, J0128, J1020, J1030, J1040, J1050, J1051, J1380, J1950, J2920, J2930, J3315, J7509, J7510, J7512, J8499, J8499, J8530, J8540, J8600, J8999, J8999, J8999, J8999, J8999, J8999, J9031, J9035, J9043, J9060, J9070, J9155, J9165, J9171, J9178, J9202, J9207, J9217, J9218, J9219, J9225, J9245, J9267, J9293, J9351, J9355, J9360, J9390, Q2043, S0138, S0165, S0175, S0179, S9560, 11900, 11900, 11900, 11901, 11981, 11982, 11983, 20600, 20605, 20610, 51720, 51720, 54520, 54522, 54530, 54535, 54690, 79101, 90471, 90472, 90585, 90586, 96365, 96365, 96366, 96372, 96372, 96372, 96372, 96372, 96372, 96374, 96402, 96402, 96402, 96402, 96409, 96409, 96409, 96409, 96409, 96409, 96409, 96413, 96413, 96413, 96413, 96413, 96413, 96413, 96413, 96413, 96413, 96415

**Figure S1**: Flow chart of study participants: men with invasive prostate cancer from the Surveillance, Epidemiology, and End-Result-Medicare data, 2004-2016, stratified by end-stage kidney disease diagnosis before prostate cancer and Black or White race


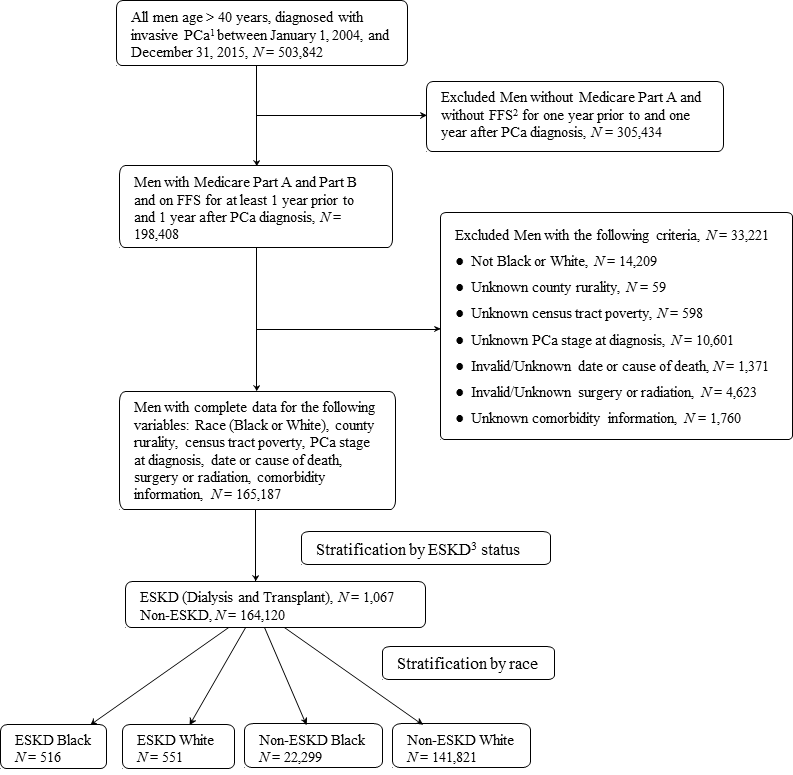


1. Prostate Cancer, 2. FFS: Fee-for-Service, 3. ESKD: End Stage Kidney Disease

**Figure S2**. Kaplan Meier curves for all-cause mortality for men with prostate cancer by race and end-stage kidney disease (ESKD) diagnosis before prostate cancer (age ≥ 66) from the SEER-Medicare data, 2004-2016. A. Men diagnosed with local stage prostate cancer. B. Men diagnosed with regional or distant stage prostate cancer.


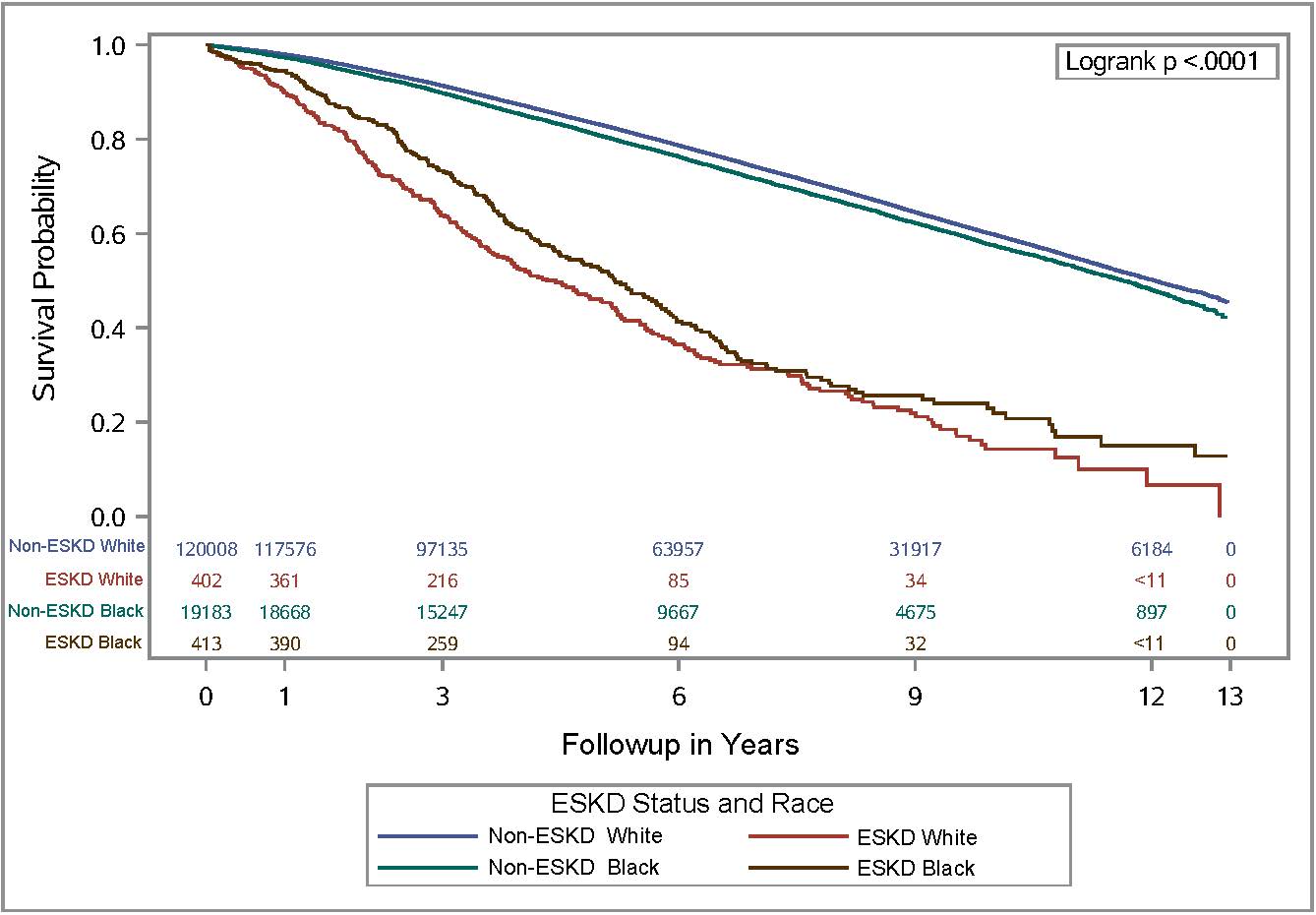


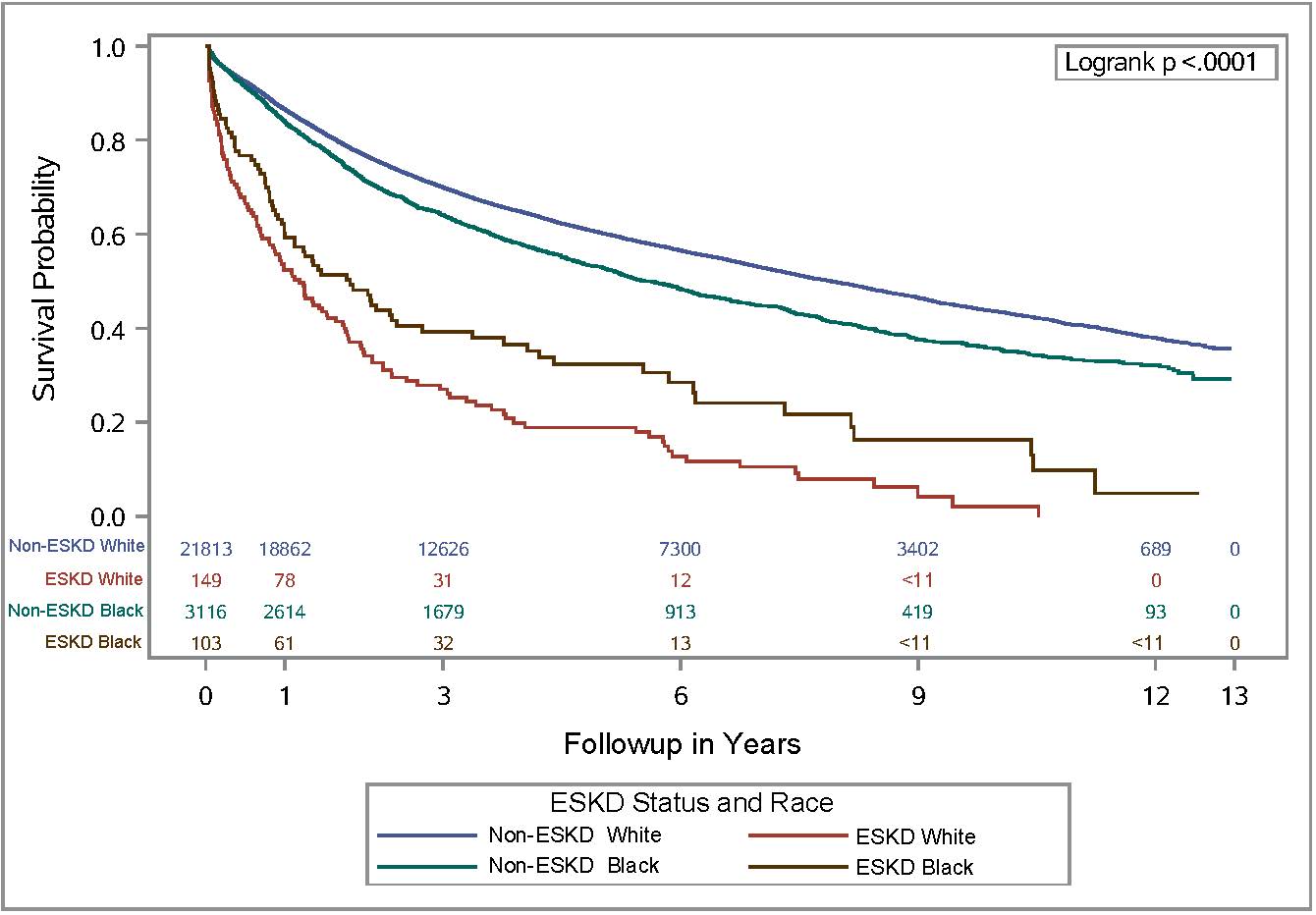


**Figure S3:** Overall mortality by stage at diagnosis of men with invasive prostate cancer from the Surveillance, Epidemiology, and End-Result-Medicare data, 2004-2016, stratified by end-stage kidney disease before prostate cancer diagnosis and Black or White race.


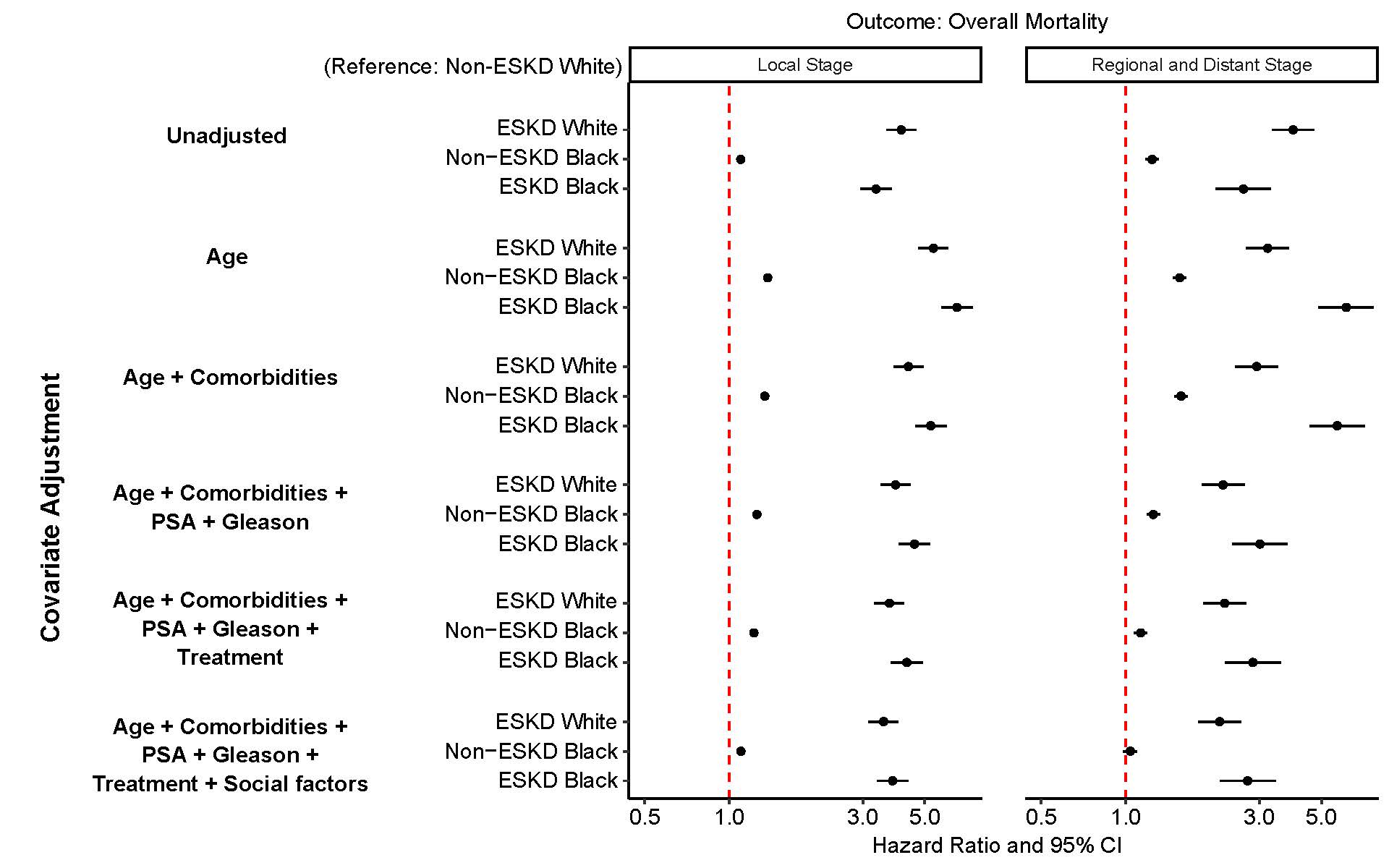

Supplement: Supplementary file 1 — Data S1. [file CAM4-13-e7027-s001.docx]
